# Mechanisims of asthma and allergic disease – 1065. Association between level of serum IL 31 and IL 33 and pruritus in Malaysian allergic patients

**DOI:** 10.1186/1939-4551-6-S1-P63

**Published:** 2013-04-23

**Authors:** Noor Suryani Mohd Ashari, Siti Nur Syuhada, AR Azriani, I Zulrushydi

**Affiliations:** 1 Universiti Sains Malaysia, Malaysia; 2 Hospital Raja Perempuan Zainab II, Malaysia

## Background

Generally allergy refers to an exaggerated immune response and a reaction that causes symptoms including pruritus in a predisposed person, which in turn can cause inconvenience or a great deal of misery. The immune responses were regulated by cytokines. This study was done to determine the association between IL 33 levels and pruritus in patients with allergic diseases.

## Methods

This was a comparative cross sectional study. Cases were patients having allergic diseases such as atopic dermatitis (AD) attending the skin clinic of Hospital Universiti Sains Malaysia (HUSM) and Hospital Raja Perempuan Zainab II (HRPZ II), Malaysia, allergic rhinitis (AR) attending ENT clinic in HUSM and atopic asthma (AA) attending chest clinic and pediatric clinic in HUSM. The patients were chosen from the history taking by the physician. The blood was centrifuged and analyzed for IL 33 using ELISA kits (Human IL 33 Duoset, RnD System). Independent T-test was used to compare the level of serum IL 33 between those with and without history of pruritus.

## Results

A total number of 210 allergic patients were included in this study. Majority of the patients were female (64.8%) and non smokers (82.5%). The result showed there was no significant difference in the mean level of IL 33 serum in non pruritic (mean of 254.71, SD of 906.80) and in pruritic allergic patients (mean of 1219.61, SD of 8660.01, p=0.156). There was also no significant difference in the mean level of IL 31 serum (p=0.241). However, the levels was higher in pruritic patients (mean of 6168.25; SD 30552.07) than in non pruritic patients (mean of 3058.09, SD 12527.97).

## Conclusions

The results of this study suggest that there was no significant association between pruritus and the level of serum IL 31 and IL 33 in Malaysian allergic patients.

